# How did primary health care in Beira experience Cyclone Idai?

**DOI:** 10.4102/phcfm.v14i1.3626

**Published:** 2022-11-01

**Authors:** Christian L. Lokotola, Tibo Uyttersprot, Priscilla Felimone, Charlotte Scheerens

**Affiliations:** 1 Department of Family Medicine, Stellenbosch University, Cape Town, South Africa; 2 Department of Public Health and Primary Care, Ghent University, Ghent, Belgium; 3 Provincial Health Service of Sofala Province, Beira, Mozambique; 4 Department of Economics, Ghent University, Gent, Belgium; 5 The United Nations University Institute on Comparative Regional Integration Studies (UNU-CRIS), Bruges, Belgium

**Keywords:** primary health care, climate change, cyclone, healthcare services, climate-resilient healthcare, adaptation

## Abstract

Beira, the biggest city of Mozambique’s Sofala province, was struck by Cyclone Idai on 14 March 2019, with devastating impacts. The floods along with the cyclone destroyed road infrastructure and health facilities and disrupted primary health care (PHC) service delivery. In addition, destruction of farmland and food stocks resulted in malnutrition; the abundance of water fostered the reproduction of mosquitos, exacerbating the burden of malaria; and problems with water and sanitation led to epidemics of cholera. The exact role and contribution of human-induced climate change is very difficult to quantify, but there is little doubt that climate change is driving more frequent and severe cyclones, such as Idai. Considering the current climatic changes, it is expected that climate hotspots such as Beira will only experience more frequent extreme weather events. In these settings, with high risks but low adaptive capacity, dedicated efforts are required to strengthen PHC with a focus on preparedness for disasters. This should entail community awareness and education, strengthening infrastructure and service provision, as well as collaboration with important stakeholders across other sectors.

**Contribution:**

Using a case study approach, this article contributes climate resilient PHC for better preparedness to service continuity.

## The context – Beira

This article reports the experiences and expertise of Dr Priscilla Felimone, complemented with available scientific literature. Dr Priscilla Felimone is a family physician, currently working at the provincial health service of Sofala province in Beira.

**FIGURE 1:**
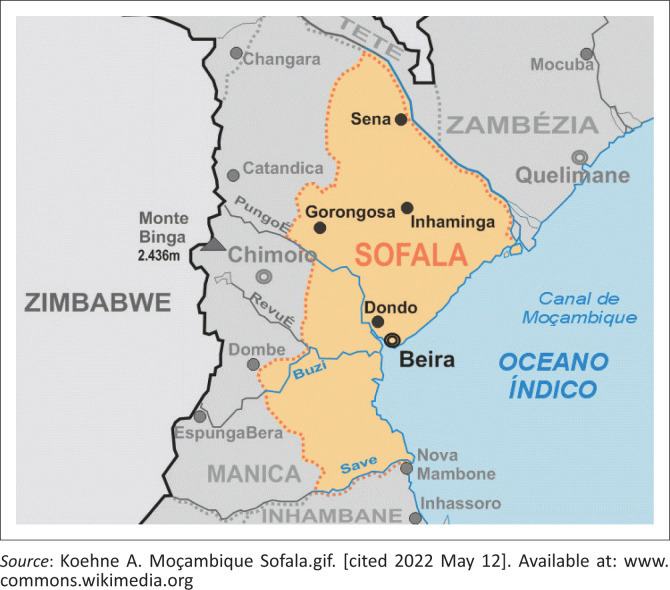
Map showing the location of Beira.

Beira is the biggest city of the Sofala province in Mozambique, situated on the coast of the Indian Ocean (Figure 1).^1^ The total population is around 530 000 people, according to the 2017 national census.^2^ The Ndau and Sena tribes are the most prominent in the area, and Ndau, Sena and Portuguese are the main languages spoken.

The health system consists of four levels of healthcare. Primary health care (PHC) comprises the first and second levels, and there are in total 176 health facilities in Sofala province. Beira has a central referral hospital (fourth level) but no provincial hospital (third level).^3,4^ This means there is no intermediate level of care in Beira, and all patients are referred from PHC to the central hospital.

In Beira there are 18 PHC centres, each responsible for a dedicated section of the population. This means that patients preferably access services at the PHC centre they are allocated to but are not sent away if they consult at a different PHC centre. All health services are provided for free, except medication, which is provided for a fixed rate. There is no distinction in service provision based on nationality. In each PHC centre, there is a variety of health professionals. At least one medical doctor is present, but large PHC centres can employ up to four doctors. Nurses, technical officers and community health workers work alongside the doctors and complete the minimum 15–20-person staff mix in each centre, but the staff count can reach up to 100.

As entrenched in the Alma Ata declaration, PHC operates on five main principles, including intersectoral collaboration.^5^ An intersectoral approach includes participation of community leaders and specific stakeholders that collaborate on early prevention, preparedness and recovery responses. In line with this, a community comanagement committee is established in each centre for early detection of health problems in the community. The focus of service provision is health promotion and disease prevention.

Under normal circumstances, there is a seasonal burden of disease in Beira. Respiratory diseases and related mortality increase during the cold season, between May and November. In the warm season, vector-borne diseases, such as malaria, are most prominent. Mortality from HIV, AIDS and hypertension are high in adults. In children, the mortality rate is highest for respiratory diseases. However, mortality and morbidity changed drastically during Cyclone Idai.

## Impacts of the cyclone and the link to climate change

When Cyclone Idai struck Beira on 14 March 2019, the entire community, including the health professionals, had very limited awareness of what could happen and were unprepared. Therefore, the effects were devastating for the entire population.

Across Mozambique, over 1000 people were killed, and in Beira about 90% of the city’s infrastructure was destroyed because of the cyclone and flash floods.^6,7^ The PHC centres had moderate to severe infrastructural damage, and the roads to access them were also badly damaged. As a result, patients and staff could not get to the PHC centres. Not only were the patients unable to attend the PHC centres, but many were afraid to go. This led to a decrease of PHC service delivery.

The usual daily patient count dropped from 200 to 300 percent to about 20–25 patients in the direct aftermath of the cyclone. As people from the middle and upper classes were better protected because of safer housing conditions, the patients who consulted the centres were often the most vulnerable people, desperately seeking health services for critical injuries. Health workers could not provide services to the best of their capacity, as the infrastructure was destroyed and transport possibilities were very limited, although routine basic service delivery was continued as much as possible in tents and field hospitals, for example from humanitarian organisations such as the Red Cross.

Many health workers had to live at the PHC centres to maintain services and were overstressed because of the increasing workload. In addition, as local people were relocated to other areas, once these were considered safe, displacement put an extra burden on the PHC centres in those safe areas.

The long-term impacts of the cyclone and floods were seen through changes in the burden of disease. The floods destroyed farmland and led to shortages of food, with an increase in malnutrition. Exemplifying the malnutrition was an outbreak of pellagra, a disease that was not present before the cyclone.^8^ Problems with sanitation and water management led to cholera outbreaks.^9,10^ Water inundation, because of the floods, created better breeding conditions for mosquitos, and as a result, malaria (already endemic before the cyclone) had an increased wave of infections.

The exact role and contribution of human-induced climate change is very difficult to quantify. However, Cyclone Idai was not at all an isolated incident, and climate change is a strong risk factor. The sixth assessment report of the Intergovernmental Panel on Climate Change, Working Group 2 (Intergovernmental Panel on Climate Change [IPCC] W2) stated that climate risks appear faster and with an increased frequency, intensity and duration of extreme weather events, including droughts, marine heatwaves, cyclones and floods.^11^ In the months before the cyclone hit, there had been a long-lasting drought and extremely high temperatures, which were intensified by climate change.^12^ As a result of this, the land was more susceptible to flooding, with water running off rather than sinking in. In addition, sea level rise because of climate change contributed to more extensive flooding. All factors combined indicate the important role of climate change on the impact of Cyclone Idai.^12,13^

## Implications for the future

Better preparedness of PHC for such disasters is essential. Primary health care must help communities to be more aware of the risks of extreme weather events and what to do. In terms of infrastructure and equipment, everything should be adapted to withstand disasters, which will ensure full operability in case of adverse events. For example, telecommunication equipment and cables should be protected to maintain the functioning of the emergency warning system; an alternative electrical power source should be available in a secure and accessible place; a permanent water supply should be available to provide 60 L of water per resident patient per day; and the medical and laboratory equipment should be protected for the impacts of disasters.^14,15^ Another key implication relates to the ongoing provision and resilience of services. This implies a need for protocols and plans of how to adapt and reorganise services in case of such emergencies. Strategies to maintain patient transport, services and supplies are fundamental and might include the availability of helicopters.^16^ Building strong intersectoral relationships and community engagement will enable collaboration to maintain services in the face of such disasters.^17^ Lastly, to ensure access to PHC, efforts are needed to prepare for the challenges related to displacement of populations and increased demands for PHC in areas receiving the population. Collecting data on PHC performance during such events and on the number of migrants entering or leaving the area would prove useful for this purpose.

## Conclusion

Considering the current climatic changes, it is expected that climate hotspots such as Beira will only experience more frequent extreme weather events. For this reason, it is argued that in these settings with high risks but low adaptive capacity, dedicated efforts such as raising community awareness, adapting infrastructure and equipment and building intersectoral relationships are required to strengthen climate-resilient PHC.
